# Addressing the Challenges of Electronic Health Records Using Blockchain and IPFS

**DOI:** 10.3390/s22114032

**Published:** 2022-05-26

**Authors:** Iris Cathrina Abacan Pilares, Sami Azam, Serkan Akbulut, Mirjam Jonkman, Bharanidharan Shanmugam

**Affiliations:** College of Engineering, IT & Environment, Charles Darwin University, Ellengowan Drive, Casuarina, NT 0810, Australia; iris.abacan@gmail.com (I.C.A.P.); s3rkanakbulut@gmail.com (S.A.); mirjam.jonkman@cdu.edu.au (M.J.); bharanidharan.shanmugam@cdu.edu.au (B.S.)

**Keywords:** blockchain, cryptography, electronic health record, privacy, security, distributed file system

## Abstract

Electronic Health Records (EHR) are the healthcare sector’s core digital strategy meant to improve the quality of care provided to patients. Despite the benefits afforded by this digital transformation initiative, adoption among healthcare organizations has been slower than desired. The sheer volume and sensitive nature of patient records compel these organizations to exercise a healthy amount of caution in implementing EHR. Cyberattacks have also increased the risks associated with non-optimal EHR implementations. An influx of high-profile data breaches has plagued the sector during the COVID-19 pandemic, which put the spotlight on EHR cybersecurity. One objective of this research project is to aid the acceleration of EHR adoption. Another objective is to ensure the robustness of the system to resist malicious attacks. For the former, a systematic review was used to unearth all the possible causes why the adoption of EHR has been anemic. In this paper, sixty-five existing proposed EHR solutions were analyzed and it was found that there are fourteen major challenges that need to be addressed to reduce friction and risk for health organizations. These were privacy, security, confidentiality, interoperability, access control, scalability, authentication, accessibility, availability, data storage, data ownership, data validity, data integrity, and ease of use. We propose EHRChain, a new framework that tackles all the listed challenges simultaneously to address the first objective while also being designed to achieve the second objective. It is enabled by dual-blockchains based on Hyperledger Sawtooth to allow patient data decentralization via a consortium blockchain and IPFS for distributed data storage.

## 1. Introduction

The world’s shift to digital technology has greatly improved the way people live, work, study, and communicate with each other. Each player in the economy is forced to keep up with rapid technological advancements; however, not all industries have the same pace of adoption. The healthcare sector is one that is slow to adopt despite large investments from governments and for a good reason [1,2,3,4,5]. This sector has one of the strictest requirements of any industry, given that it deals with human lives.

The advantages of digitalization, such as efficiency, scalability, flexibility, reliability, and cost-effectivity, are difficult to dispute [6,7,8]; however, because the matter of safety is the top priority in healthcare organizations, they cannot risk adopting immature technology [9,10]. This does not mean that the healthcare sector has not been part of the ongoing digital transformation, but they are often laggards in terms of adoption. There is one system central to health care that has quickly become the core of the sector’s digital strategy–health records [11]. The desire to continually provide quality care to people with the aid of technology has led to the conception of EHR. It contains sensitive information and, in many countries, this is protected by law via the Health Information and Portability Accountability Association [12].

EHR intends to accurately consolidate every patient’s record from different healthcare providers over time [13] and then ultimately show the health status of a patient just by checking their own EHR. In addition to this, its adoption was thought to bring a remarkable improvement in the traditional healthcare system by lowering medical errors, improving healthcare quality, reducing healthcare costs [14,15,16,17], and providing health education to encourage conscious decisions about health care [18].

Going electronic is the way forward but this makes it very susceptible to cyberattacks [12,19] and this has become a fundamental challenge in the current setting [20]. Privacy and security are two of the main concerns of the public [21,22,23,24], and their trepidations are totally valid. Privacy, or information privacy, to be more specific, refers to a patient’s personal information that is collected to help identify an individual [23]. Security, on the other hand, is about restricting and authorizing access to personal information [25]. Both of which are essential as leaked information may lead to serious consequences.

The world has seen how their information can easily be mishandled as cyberattacks against hospitals are continuously rising. This worsened when COVID-19 suddenly hit the world. Issues that require immediate attention have been exposed and have led to serious problems such as disrupted services, expensive payments to ransomware attackers, compromised health records being sold on the black market, and availability of essential health care services [26,27]. The big data that the healthcare sector holds has become a lucrative source for ransom and attackers are taking advantage of this pandemic. Respective officials and authorities have been working hand in hand to address these issues.

Researchers have been exploring ways to solve these issues [28] way before the pandemic. Numerous proposals have been published and most of the solutions proposed point toward addressing privacy, security, data ownership, access controls, and data storage using cryptography [24,29,30]. There have been a growing number of attempts domestically and overseas to apply blockchain technology in the healthcare industry [17] to address EHR issues. This paper reviewed and evaluated these existing proposals side by side to summarize their findings and to determine the current state of research on this topic. Based on the results of the conducted review, a new framework was proposed using a dual-blockchain based on Hyperledger Sawtooth. This allowed patient data decentralization via a consortium blockchain and IPFS for distributed data storage.

The rest of the paper is organized as follows. Section 2 discusses the systematic review conducted to find the gaps in this research area and to determine the basis of the framework to be proposed. Section 3 shows the results of the systematic review, which revealed the challenges that currently exist in EHR. The framework is then designed and proposed based on these challenges. Section 4 is about discussion and tackles and interprets the results. Lastly, Section 5 concludes the paper and discusses its future direction.

## 2. Materials and Methods

The foundation for creating the ideal EHR solution requires establishing a state-of-the-art for EHR, so it is beneficial to evaluate previous proposals and attempts. This allows system architects to figure out if there are still gaps that need to be addressed and if they can be filled by a different solution. Blockchain is one of the most promising technologies being explored [31], and this study looks at blockchain-based solutions that aim to solve the challenges of EHR.

Blockchain has inherent properties that provide privacy and security to data that were the primary concerns for EHR adoption, thus making it a front-runner in the race to create the perfect EHR system [32]. Aside from those advantages, this technology also brings system durability, robustness, and availability due to its decentralized and distributed nature. With the right design and implementation, it is theoretically possible to secure healthcare records against the increasing threat of ransomware and data breaches while achieving the dream of wrestling control of healthcare data from organizations and giving them back to patients.

Another important factor is how the EHR data are stored and what options are available that would meet the research objectives. Traditionally, on-premises storage has been the preferred choice to ensure the safekeeping of sensitive patient data. Recent advancements in cloud offerings have seen a shift to storing more and more patient data in remote datacenters. Today, IPFS is another option that has been investigated for use with EHR.

### 2.1. Systematic Review of Existing Proposals to Solve EHR Issues

According to Kitchenham [33], an appropriate approach to gathering existing studies and determining the gaps that may suggest a new area of research is by conducting a systematic review. To obtain a summary of existing blockchain-based solutions that aim to solve the existing challenges of EHR, a systematic review was performed. The review protocol is split into two major steps: (1) the search phase and (2) the specifications of inclusion and exclusion criteria.

The search phase includes defining academic databases, digital libraries, and search engines that can be used to search for eligible studies and then determining queries to be used to find all related results by using AND/OR Boolean operators. Table 1 shows the databases used for this systematic review. Table 2 lists all the search queries used.

The next phase is specifying the inclusion criteria (IC) and exclusion criteria (EC). These were carefully set out in order to refine search results. Table 3 shows the list of IC and EC for this study. All studies that fell under EC were deemed ineligible right away. Title, abstract, and full text of studies were screened further to obtain a more refined search result.

(a) Title: papers that did not match at least one of the keywords listed in Table 2 were eliminated.
(b) Abstract: papers that satisfy at least 40% of IC were retained for further screening.
(c) Full text: papers should discuss proposals that address EHR challenges.

**Figure 1:**
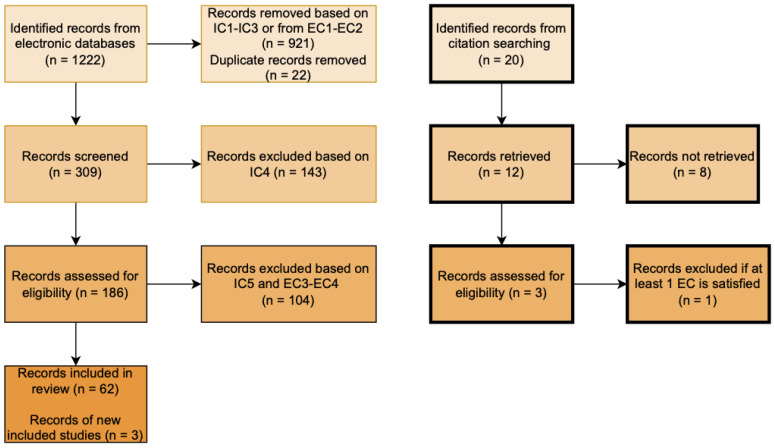
Systematic review flow diagram.

A total of 1242 records were obtained initially, of which, 1222 were obtained from database search and 20 obtained through other sources. Results were reduced to 309 from database search and 12 from other searches. After applying all the eligibility criteria, 62 studies were left from the database search and 3 from other sources. A total of 65 studies were found eligible for this paper. This process is better shown in Figure 1.

### 2.2. Quality Assessment of Systematic Reviews

To ensure the papers included are trustworthy and suitable for this systematic review, the quality of the papers is assessed using two methods. The first method adapted from previous SLRs [34,35,36] is applied by setting quality evaluation (QE) questions. Each of these QEs are answerable by no (0), partially (1), or yes (2). There are five questions and each one can be marked 0, 1, or 2 per reviewer.

The score for each question is a cumulative score of two markers; thus, 4 is the maximum score for each. There are five questions in total, so the highest grade per publication is 20. Both the reviewers decided that a cumulative score between 0 to 10 would be considered a failure, which means the paper has to be excluded from the review. A cumulative score of 11 to 20 is a pass, which means the paper is very suitable to be included in the review. Table 4 shows the summary of the conducted quality assessment of papers included in systematic review. 

**Figure 2:**
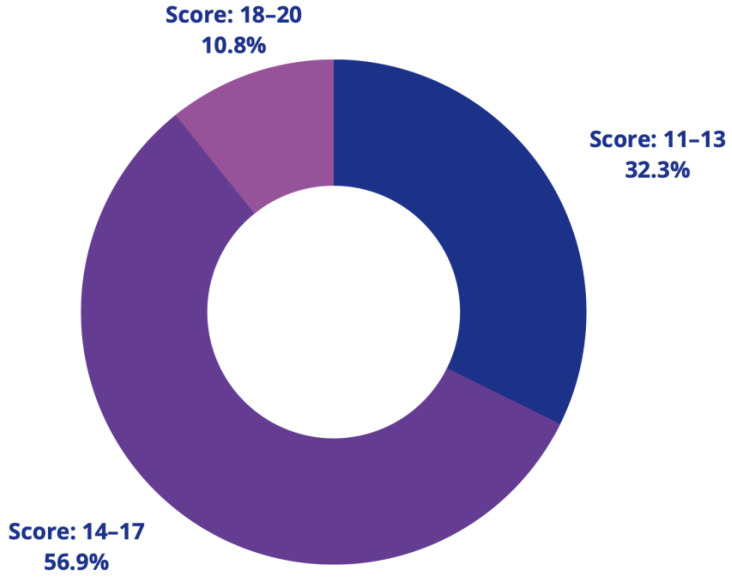
Result of quality assessment of 65 papers.

Based on the results, all 65 papers have passed the threshold mark and are included in the review. Figure 2 shows the results in a snapshot.

**Figure 3:**
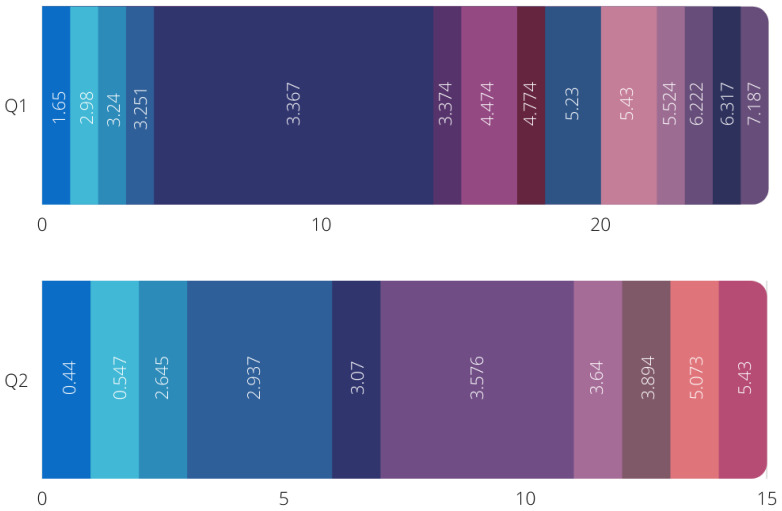
Distribution of Q1 and Q2 journal and their impact factor rating.

**Figure 4:**
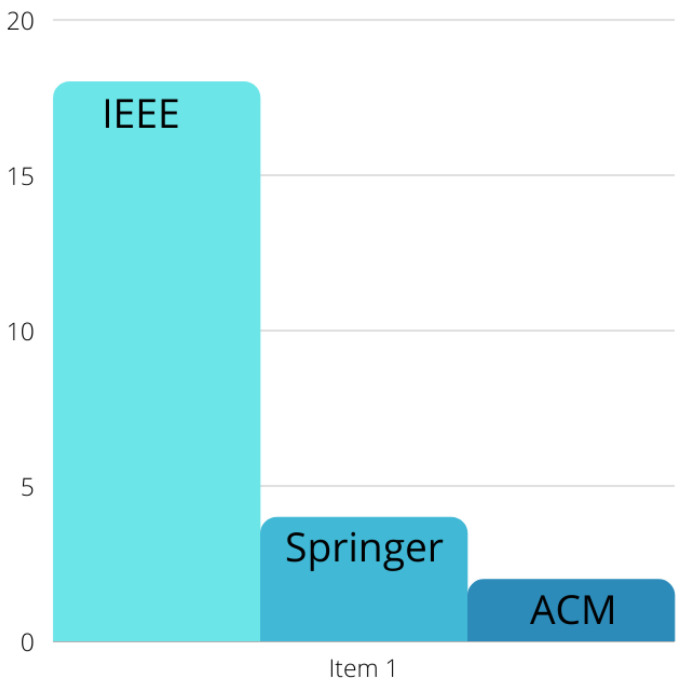
Distribution of conference papers.

The second method is ensuring the quartile of the papers are within Q1 and Q2 and that their impact factors are of a good ranking. Of the 65 papers included in this systematic review, 26 papers were from Q1 journals, 15 papers were from Q2 journals, and the remaining 24 papers were from IEEE, Springer, and ACM conferences. Figure 3 shows both the Q1 and Q2 distribution of journals with their impact factor ratings. Figure 4 shows the distribution of conference papers.

### 2.3. Summary of Proposed Solutions

Table 5 lists 65 blockchain-based solutions that were proposed in an attempt to solve issues that exist in EHR. Data extracted from all of the papers were carefully analyzed, from the type of blockchain that was used, the technologies that were utilized for their design, the status of each proposed solution, whether it was a proposal in theory, simulated, or implemented, and the challenges that each paper tried to address. There were multiple challenges addressed across each of the 65 novel solutions and these demonstrate the areas of focus that are broadly regarded as important to realizing the benefits of successful implementation of EHR.

**Figure 5:**
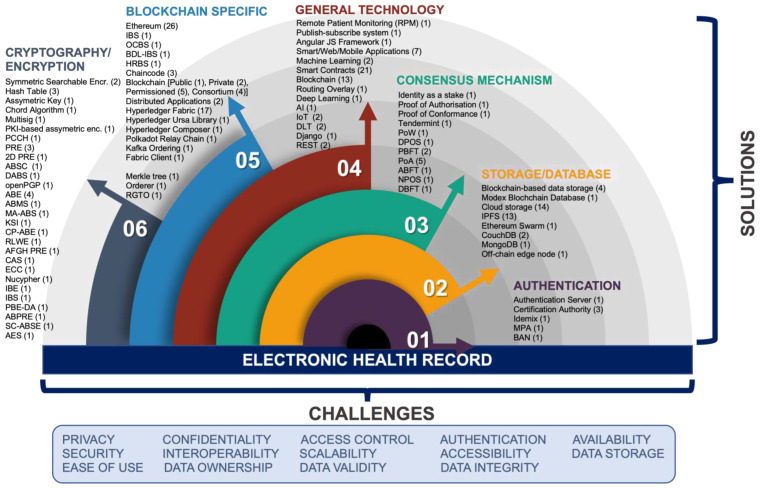
EHR challenges and the technologies used as solutions.

Each proposed solution was carefully studied to identify the challenges that were explicitly being addressed and these were recorded and summarized into 14 main challenges as shown in Figure 5: privacy, security, confidentiality, interoperability, access control, scalability, authentication, accessibility, availability, data storage, data ownership, data validity, data integrity, and ease of use.

Figure 5 also sums up all the technologies used across all papers that were recorded. The frequency of use of each technology in the proposed solutions is recorded and shown in numbers enclosed in parentheses next to each of them. It was found that some technologies are prevalently used, such as Ethereum, Hyperledger Fabric, blockchain (general/not specified), smart contracts, cloud storage, and IPFS. All the technologies used can be fit into 6 categories, including: general technology, blockchain-specific, consensus mechanism, cryptography/encryption, storage/database, and authentication.

### 2.4. Challenges Addressed by Proposed Solutions

**Figure 6:**
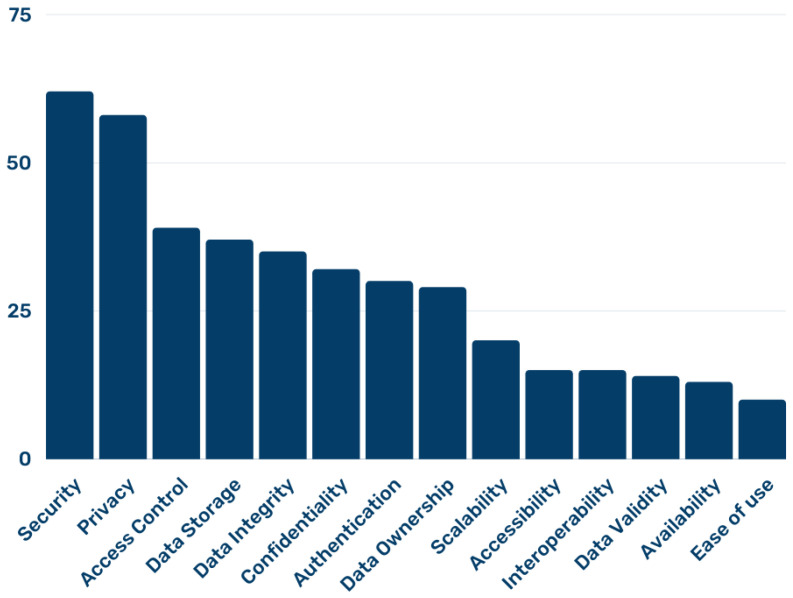
Number of papers addressing each EHR challenge.

Figure 6 shows the number of papers that tried to address each EHR challenge in their proposed solutions. Out of the 14 challenges cited across all publications, security was the top-most concern, with 63 publications listing them as a focus. Privacy came second, with 59 publications citing it as an area of concern. Access control came third with a significant difference as only 42 papers made it explicit that their solution was focused on it. Not far behind access control is data storage, data integrity, confidentiality, authentication, and data ownership. Ease of use faced challenges, as it had the least number of papers highlighting it as a major focus.

#### 2.4.1. Security

Blockchain is mainly considered for EHR for its intrinsic ability to provide security and data immutability via decentralization and cryptography [102]. For example, PESchain proposed and simulated by Jiang, S., H. Wu, and L. Wang [72] is a consortium blockchain maintained by a set of medical institutions whereby EHR data are encrypted and stored in the local cloud while only their hashes are added to the blockchain. A novel solution was proposed by Wang, H. and Y. Song [53] that utilized Attribute-Based Encryption (ABE) together with Identity-Based Encryption (IBE) to encrypt data ensuring fine-grained access control and implementing Identity-Based Signatures (IBS) for digital signing. To achieve security in their system, they introduced a new primitive called combined attribute-based/identity-based encryption and signature (C-AB/IB-ES). Another approach is displayed in SHEMB proposed by Andola, N. et al. [55], which uses the Ethereum blockchain to store EHR data and utilizes smart contracts [52,54,61,66,91] to securely give patients control over their data. Searching for patient data is made more secure and efficient with the use of symmetric searchable encryption.

#### 2.4.2. Privacy

Privacy is one of the major concerns in EHR systems implementations. Blockchain tackles this issue by employing encryption techniques to obfuscate the link between patients and the confidential data that relate to them. For example, in the paper by Guo, R. et al. [94] privacy of patients is preserved by employing Multi-Authority Attribute-Based Signatures (MA-ABS), which require users to sign messages with any predicate of their attributes issued by multiple attribute authorities that harden the security compared to single authority ABS protocols. Another approach was suggested by Stamatellis, C. et al. [74], where an identity mixer system used in conjunction with Hyperledger Fabric is used to hide the identity of patients. Idemix is an extended anonymous credential system developed at IBM Research, which comprises attributes, credentials, and optional components, namely commitments and pseudonyms. A credential contains a set of attributes that comes with cryptographic information [103]. Search encryption and proxy re-encryption technologies were combined by Wang, Y. et al. [46] and implemented on Ethereum with the assistance of cloud storage [54,60,70] to realize privacy preservation and data security. Only the authorized data requesters who have a searching trapdoor are allowed to acquire the keywords and related information using the blockchain.

#### 2.4.3. Access Control

Access control is closely related to data ownership that describes the selective restriction of information and systems so that only authorized users can have access. Thwin and Vasupongayya [41] proposed a blockchain-based model that has fine-grained access control of medical records [43,53] stored on cloud storage and uses proxy re-encryption [47] for data sharing. The actual medical files are encrypted using the owner’s public key, while the re-encryption keys and other information needed in the authorization process are stored on a semi-trusted proxy called the gateway server. A group of authorized users, according to an access list, can access the ciphertext of the medical records using the re-encryption keys, which are granted and revoked by the data owner. OSHealthRec, which is a blockchain-based prototype by Meier et al. [45], also offers fine-grained access control that enhances data privacy and security. Data owners can revoke access authorization at any time or grant one-time access only. All processes regarding access management are carried out on the blockchain and thus are logged in a transparent and traceable way. They used the hyperledger composer to model the network participants and assets using a unified modeling language (UML) class diagram and implemented functions as transactions on the blockchain.

#### 2.4.4. Data Storage

Along with the expected increase in data, it is foreseen that there will also be a rise in the need for cost-effective data storage. Reen et al. [73] proposed a mechanism that uses a permissioned Ethereum blockchain with a combination of symmetric and asymmetric key cryptography to ensure secure data storage with IPFS. A smart contract is used to store the mapping between the patient/combined key and the IPFS hash values. Another smart contract is employed so that patients control the granting and revoking of access. A different proposal was made by Sun et al. [87] that described a holistic on-chain and off-chain collaborative storage system for the efficient storage and verification of EHR data [58]. This meant that only the addresses of the EHR data are stored on the blockchain, which has limited storage capacity and computational resources. The actual EHR data are encrypted and stored on participant nodes instead. El Sayed and Abdelaziz [40] take this a step farther in terms of efficiency and performance by utilizing a hash table to store and fetch medical data from the cloud. The EHR data are encrypted using the blockchain’s hash function to maintain confidentiality and a digest of the data is generated after hashing. This digest is then used as a key in the hash table to create the index referring to the actual location of the EHR data.

#### 2.4.5. Data Integrity

With the use of both centralized and decentralized forms of storage, data integrity has risen as another noteworthy issue that the considered proposals aimed at addressing. Hyla and Pejas [83] discussed a blockchain-based eHealth integrity model (BEIM) for ensuring information integrity for a permissioned blockchain with off-chain information storage. In their proposed model, a blockchain is mainly used to implement a data-integrity service. A user’s public key is used to encrypt blockchain transactions, while decryption using the private key is given by the central eHealth service to an entity that has an EHR integrity-verification access right. After verification, the private key is destroyed by the verifier’s application. In Healthchain [37], Chenthara et al. employed special public-key cryptography for encrypting the data in the IPFS off-chain storage [81]. The system allows only the permissioned or authenticated users to access the record for a particular session. Patients have complete control of granting and revoking access to their medical records to preserve the confidential nature of health data [41,47,60,67,81].

#### 2.4.6. Confidentiality

Another issue tackled in the eligible papers that is related to privacy is confidentiality, which deals with keeping the information of users from being divulged to the public. Benil and Jasper [68] proposed a new scheme named Elliptical Curve Certificateless Aggregate Cryptography Signature scheme (EC-ACS), which enhances confidentiality through encryption of medical data being stored on the cloud. The encryption makes it difficult for malicious agents to forge the medical data blocks, and the secure certificateless public auditing scheme allows the checking of data using an auditor that is integrated into a transaction on the blockchain. In another paper that preferred the use of cloud storage, Yang et al. [48] proposed BVOABSC, which combines an attribute-based signcryption (ABSC) algorithm and the blockchain technology that allows the confidential sharing of medical data in a multi-authority cloud storage environment. Their scheme ensures that the size of the ciphertext is constant, thus hiding the number of attributes and giving the ability to implement flexible access control confidentially. Demir and Kocak [75] proposed a general framework for sharing critical documents not limited to the EHR domain using blockchain and IPFS. Because the medical files would be stored on public IPFS nodes, the system will use access control and encryption to ensure the confidentiality of the health records [81,101].

#### 2.4.7. Authentication

Authentication is one of the challenges in the list that is related to security and access control that did not receive a lot of focus but is crucial to the robustness of the proposed EHR solutions. Li, C.T. et al. [79] proposed a data aggregation scheme and group authentication based on blockchain technology. Multiple authorized users such as patients, doctors, caregivers, family, and friends can freely access a patient’s personal health records [48,71] using a group session key. Upon mutual authentication and joint agreement, this key can then be used by group members to view the encrypted private information. In contrast, Alruwaili [80] went with a novel approach of using artificial intelligence and a multi-agent-based distributed ledger system to improve EHR security. Intelligent multi-agents for user authentication agents are established as well as for providing access to EHR datasets. A user interface is used to accept username and password, which are checked by the authentication agent to validate the entry of the user. This agent makes an effective comparison of already stored datasets and the providers willing to access the datasets by issuing a digital certificate based on blockchain technology.

#### 2.4.8. Data Ownership

Data ownership was also a focus in the proposed solutions to alleviate fears expressed by EHR users regarding third parties accessing their confidential healthcare data. Solutions to this issue also work well with the encryption schemes employed in blockchain systems. Reen, G.S. et al. [73] proposed a system that requires patients, hospitals, and insurance agencies to be part of a permissioned blockchain network and IPFS for file storage [37,58]. Patients will need to grant access to their EHR when requested by hospitals. Another proposal by Fatokun, T. et al. [91] utilizes Ethereum and smart contracts so that healthcare providers can search for patients’ data and request their consent to access the data. In the KSI blockchain proposed by Nagasubramanian, G. et al. [44], doctors provide their ID and the patient’s private key when requesting health information to ensure patients maintain ownership of their data.

#### 2.4.9. Scalability

If EHR implementations succeed at being interoperable, thus allowing network effects to grow, then systems that empower them must be scalable and efficient to handle the influx of data. MedBloc, a blockchain-based secure EHR system for sharing and accessing medical data, proposed by Huang et al. [38], will use a permissioned blockchain network employing a state machine replication-based consensus mechanism. Hyperledger Fabric was chosen as the platform and a PBFT algorithm [83] was utilized instead of the slow proof-of-work consensus used in cryptocurrencies such as Bitcoin; however, Bhattacharya et al. [71] took a different approach with BinDaas, a blockchain-based deep-learning-as-a-service for Healthcare 4.0 applications. They proposed taking advantage of distributed AI [85] to address the scalability limitations of mined transactions as well as cost and storage issues of EHR. Their contribution centered around deep learning prediction models on stored EHR to reduce the feature extraction time and automate recommendations.

#### 2.4.10. Accessibility

As more EHR systems are implemented and adopted, it is important to keep in mind the users who will interact with these systems daily. Accessibility is an important objective especially in healthcare, where stakeholders are mostly non-technical. Xu et al. [86] proposed a Credible Blockchain-based E-Health Records (CB-EHRS) platform that describes the use of a user interface layer to display data and receive user input [54,104]. This layer provides a graphic interface that can interoperate with the entire blockchain and is accessible on mobile devices [79], PCs, and smart wearables. Choosing a different approach, Jain et al. [92] utilized machine learning algorithms to make the health care data accessible to researchers and doctors for the purpose of research. A dual blockchain structure was used where the first part grants access to the health data and is built on top of Hyperledger Fabric. The second part of the structure utilizes Ethereum, which performs all applications and services. Miyachi and Mackey [58], on the other hand, came up with the hybrid Off-Chain Blockchain System or hOCBS that attempts to address accessibility by focusing on the integration with current healthcare information as opposed to re-implementing core components in existing EHR systems. They intended to achieve this by combining smart contracts, digital wallets, and tokens on their application and features layers.

#### 2.4.11. Interoperability

One of the original challenges that EHR was meant to solve is unlocking siloed healthcare data to increase efficiency via data sharing between different providers. Due to different EHR implementations, medical data among them are commonly not interoperable hindering data exchange. Hylock and Zeng [47] attempted to resolve this using their blockchain framework for patient-centered records and exchange. They recommended that health information systems should conform to nationally recognized standards such as HL7 Fast Healthcare Interoperability Resources (FHIR). This standard, together with proxy re-encryption and smart contracts, form the foundation of the proposed exchange model. Roehrs et al. [69] also proposed OmniPHR, which is a distributed model that allows patient data to be interconnected between health organizations. They proposed a personal health record (PHR) representation, organized hierarchically, encrypted, and distributed in chained data blocks on the network. These blocks can be in different healthcare organizations and even in a patient-managed repository.

#### 2.4.12. Data Validity

Data validity is another important aspect of EHR systems; as the size of the data collected over time increases, so do the potential errors, which are dangerous in the healthcare industry. In BVOABSC, Yang et al. [48] implemented a cloud server [68] that verifies the validity of the ciphertext generated from healthcare data. If the ciphertext is invalid, the Cloud Server can refuse to store it. The system also allows auditors to verify the correctness of the EHR data and check the validity of letters of authorization [46]. In Benil and Jasper’s [68] (EC-ACS) scheme, a user’s private key is used for validating the signature on each transaction and the public key checks the generated signature by the private key.

#### 2.4.13. Availability

Lastly, availability is a critical challenge in systems meant for healthcare organizations that did not receive as much attention as it should have. Kalaipriya et al. [66] indulged in investigating this challenge by leveraging blockchain technology. They concluded that using smart contracts, cryptography, distributed applications, and blockchains allow EHR systems to be highly secure and available. Nguyen et al. [54] proposed a novel EHR sharing framework that combined blockchain and IPFS on a mobile cloud platform with availability in mind. This is accomplished with the help of the robustness of IPFS, which distributes the data across nodes making sure there is no single point of failure. The use of a mobile app allows users to interact with the system in a real-time and dynamic manner [104] with highly available medical data on the cloud. The model proposed by Mahore et al. [42] partnered blockchain technology with cloud storage [39,40,41,48,70,84,94] as they believe cloud technology provides a reliable storage system that helps mitigate issues with availability.

#### 2.4.14. Ease of Use

A key challenge with any system meant to be implemented in the healthcare industry is the ease of use, as a majority of users are not technically inclined. PatientDataChain proposed by Cernian et al. [56], is a patient-centered model where patients are the owners of their medical data. The core of PatientDataChain is the Patient Health Wallet app, which makes it convenient for patients to access and control their healthcare data. The patient will also be able to search for the drugs prescribed in the system as well as make online reservations. On the other hand, Mahore et al. [42] proposed a model that makes it easy for researchers to perform statistical analysis of healthcare data while maintaining privacy. This was achieved by partitioning the patient medical data into sensitive and non-sensitive. The classification helped in reducing computation time and resources, thus making healthcare metadata more accessible to researchers for obtaining statistical information.

## 3. Results

To determine the current state of research focused on proposing solutions to EHR issues, a systematic literature review was performed. Blockchain has been one of the most explored technologies in addressing EHR issues, so the studies included were all blockchain-based solutions. This review helped identify the gaps that still need to be addressed and has determined the need to propose a framework that can effectively address the issues that may be found.

Based on the systematic review, no one has proposed a solution that addresses all the challenges that currently exist in EHR; therefore, designing a framework that can address all the challenges identified is needed. There were 14 challenges, and this section discusses the technologies and techniques to be used.

The framework was consciously designed to offer a solution for each challenge to achieve the goals of this study. Based on the 65 papers reviewed, there are a lot of technologies that can be used to address the identified challenges. Hyperledger Fabric came up quite a few times but based on further research, Hyperledger Sawtooth is more applicable for the design. For storage, IPFS and cloud solutions were at the top of the list. For our framework, IPFS is used. The use of these technologies is justified in the next section.

Table 6 lists the summary of all the identified challenges together with the proposed solutions that is used to tackle each one of them. Notice that Hyperledger Sawtooth, IPFS, and the dual-blockchain system will be the basis of the framework design, but this will be complemented with other technologies and techniques to ensure all the challenges will be addressed.

### 3.1. Proposed EHRChain System Architecture and Design Principles

To achieve this, this study proposes a consortium blockchain architecture that is based on Hyperledger Sawtooth that makes use of IPFS as well as the dual-blockchain system to store and manage EHR data across consortium members.

**Figure 7:**
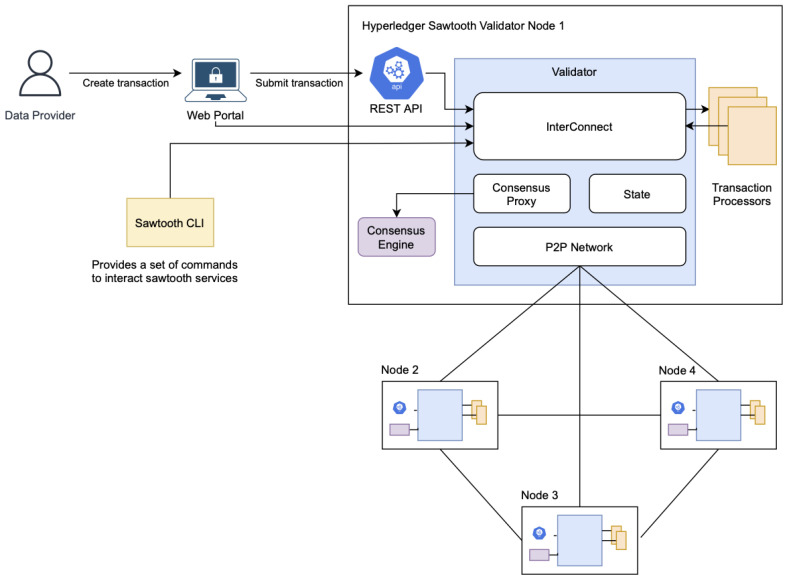
Hyperledger Sawtooth as the basis of the framework.

Hyperledger Sawtooth, as seen in Figure 7, was chosen as the basis for this framework because of the following advantages it offers:

- Allows public or private blockchain (Hyperledger Fabric can only do permissioned blockchains);
- True immutability (Hyperledger Fabric only has partial immutability);
- Flexibility—the core is separated from the application layer;
- Permissions system allows the specification of authorized validators and peers;
- PBFT consensus secures against up to 1/3 malicious validators (Hyperledger Fabric is only crash fault tolerant).

**Figure 8:**
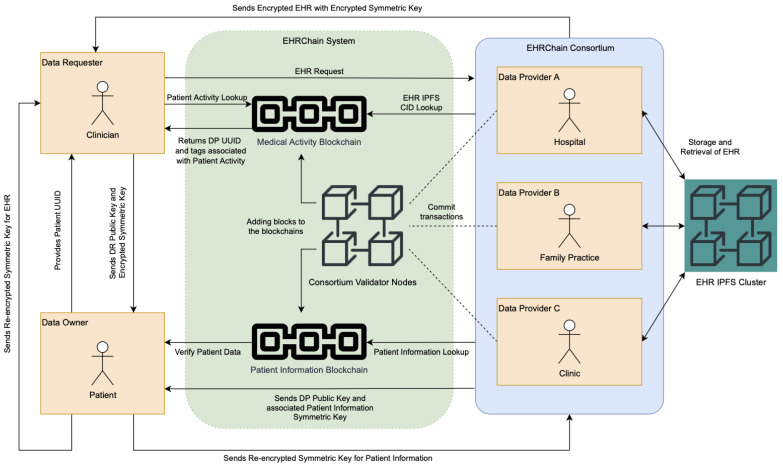
EHRChain system architecture.

EHRChain’s dual-blockchain architecture allows the separation of EHR into two independent distributed ledgers: the Patient Information Blockchain and the Medical Activity Blockchain. Partitioning them enables different security models to be applied for the two types of data allowing medical data to be shared with other organizations and researchers while protecting sensitive and confidential patient information. On the other hand, utilizing IPFS for storing EHR files avoids a single point of failure and provides a robust backup and restores the system in case of cyberattacks. Figure 8 illustrates the EHRChain system architecture using Hyperledger Sawtooth together with IPFS and a dual-blockchain system and shows how they work together to ensure all the challenges are addressed accordingly.

Together with the system architecture illustrated above, the number of system rules are specified to provide governance to the system actors and ensure that the proposed solutions corresponding to the 14 EHR challenges are properly implemented:

1. DP is required to be part of the consortium and provides one validator node and at least one IPFS node.
2. DR can either be a member of the consortium or not.
3. Patient information, medical activity, and health records are stored separately to ensure privacy.
4. Health records must be stored with at least three DP IPFS nodes, chosen randomly, for redundancy.
5. Patient information and related EHR data are encrypted with a generated symmetric key from the app, which is then encrypted with the DO’s public key DOPk.
6. Patient information is stored in separate key–value pairs to allow fine-grained access control.
7. Medical activity data are encrypted with generated symmetric keys from the web portal, which are then encrypted with DP’s public key DPPk.
8. Medical activity records are tagged with a DP’s address and medical keywords.

### 3.2. EHRChain Workflow

**Figure 9:**
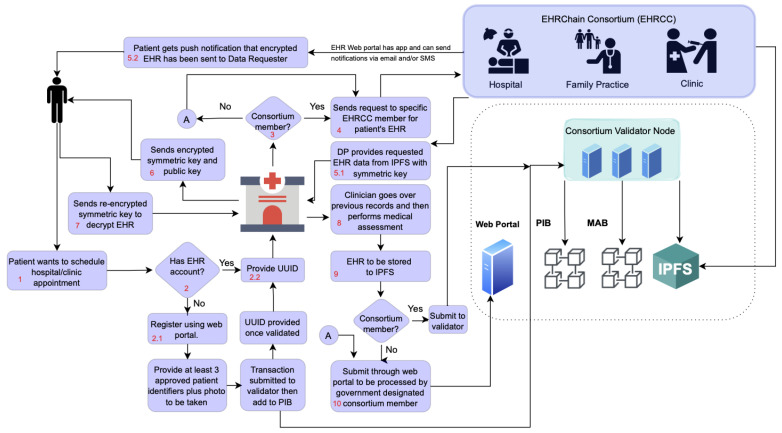
Proposed EHRChain system workflow.

The EHRChain system workflow is shown in Figure 9 and to easily follow the sequence, each step is numbered in red text. This system workflow has three types of users: DO, DP, and DR. DOs are consumers of medical services, which generate medical activity with associated health records. DOs also provide patient information, which are data that relates directly to a patient and should be private and confidential. All the information which are stored on the Patient Information Blockchain (PIB), as well as EHR stored in IPFS, are owned and administered by the patients themselves.

On the other hand, DPs are health organizations that are members of the EHRChain consortium and are in charge of committing and retrieving health records to and from the MAB and IPFS clusters. DPs only have access to the blockchain records in which they have direct involvement. These include medical appointments by patients made with doctors under that DP, as well as medical files such as scans generated during medical procedures that need to be stored on the IPFS cluster. A DP must be able to track where these files are stored and with which patient they are associated so that they can retrieve and provide them when the patient or a DR submits a request to retrieve them for whatever reason.

As alluded to, a DR is either a health organization such as a clinic or an individual practitioner that is dealing with a patient whose EHR resides on the EHRChain and requires the records to provide the best care possible for the patient. A DR could either be a member of the consortium or not. The main difference is that a DR was not involved in some of the records owned by the patient and thus, could not use their private key to access the data. When a DR requests for EHR, the DP sends the encrypted EHR together with its encrypted symmetric key. Then, the DR sends the encrypted symmetric key to the patient together with their public key to be re-encrypted by the patient and then sent back to the DR.

The workflow of the proposed EHRChain system using Hyperledger Sawtooth is as follows:

- Patient schedules an appointment at a new clinic instead of his/her regular medical provider.
- Patient arrives at the new clinic during the scheduled time.
- Clinic asks if the patient is already a user of EHRChain.
- Patient answers ‘Yes’ and provides their Patient UUID into the application.
- To access the MAB, the new clinic could either download/update their copy of the MAB, which is publicly accessible, or access it only via a proxy provider.
- Clinic searches the MAB for any medical activity records that include the UUID provided by the patient together with keywords relevant to the patient’s visit.
- Clinic retrieves records of DP UUIDs of medical organizations that the patient has interacted with before with keywords that match those that the clinic provided.
- Clinic acts as the DR and sends secure requests to the DPs for records of a given patient’s UUID via the application, as well as a list of pre-existing conditions.
- DP retrieves the relevant encrypted records from the MAB and the corresponding encrypted symmetric keys for each record.
- DP decrypts the symmetric keys using their private key and uses them to decrypt the MAB records to check if any EHR is related to the request.
- If yes, DP searches for the CID from the IPFS cluster to retrieve the encrypted EHR records together with their corresponding symmetric keys.
- DP retrieves the DR’s public key and uses it to re-encrypt the symmetric keys, then sends them together with the encrypted MAB records and encrypted EHR to the DR.
- DR decrypts the symmetric keys using their private key and then uses the symmetric keys to decrypt the encrypted MAB records.
- If the DR’s request includes any data related to patient information, they search these on the PIB and send the encrypted records to the DR together with the corresponding encrypted symmetric keys.
- Upon receiving the encrypted patient information records, the DR sends the encrypted symmetric keys together with their public key to the patient for re-encryption.
- This is also the case for the encrypted EHR files whose symmetric keys are encrypted by the patient’s public key.
- After verifying the DR’s public key, the patient re-encrypts the symmetric keys and sends them back to the DR.
- DR decrypts the symmetric keys using their private key and then uses the symmetric keys to decrypt the encrypted PIB records.
- The clinic then prepares the records for the doctor that will provide medical services to the patient. The doctor uses these records to gain context of the patient’s medical history.
- After the appointment, the doctor might have generated a new EHR that should be recorded.
- Assuming that the clinic is a member of the consortium, it will first process the data into the required data structure, encrypting the activity details and activity findings using generated symmetric keys, which are then encrypted themselves using the clinic’s public key now acting as a DP. Data structures are explained further in a separate section.
- If there is a content heavy EHR created, such as scans, this is first encrypted using generated symmetric keys, which are then encrypted themselves using the patient’s public key generating the CID and are then saved in its own IPFS cluster node. The CID is included in the activity findings data.
- Pseudo-random numbers are generated from the CID to choose two other IPFS nodes in the network belonging to other DPs that would act as redundancy.
- A request will be sent to the DPs chosen to pin the CID in one of their IPFS cluster nodes.
- A record of these IPFS nodes is submitted to the MAB so that in case of a ransomware attack, the clinic can locate where the redundant CIDs reside and retrieve them.
- The Clinic then submits the details of the appointment to the pool of medical activity records by broadcasting the activity/transaction to the validator network.
- The currently assigned block producer will then eventually take this submitted activity/transaction, group it with other activities/transactions into a block and add it to the MAB.

The view of the system workflow can be simplified by breaking it down into three distinct phases:

1. Patient registration and information retrieval;
2. EHR request and data dispensation;
3. EHR creation and blockchain storage.

#### 3.2.1. Patient Registration and Information Retrieval

**Figure 10:**
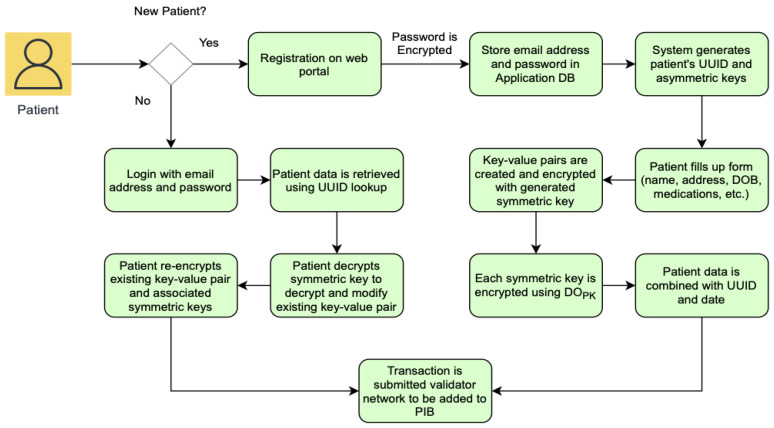
Patient registration and information retrieval flow diagram.

Figure 10 shows the flow diagram for patient registration and information retrieval. Patients are the system users that generate and own EHR as they receive medical services over time, which are recorded as medical activity on the MAB. In contrast, the patient’s information that would be stored on the PIB would be mostly static because it includes data such as the patient’s name, date of birth, etc. The patient’s information would only require infrequent modifications in case of changes, such as the patient moving to a different address.

**Figure 11:**
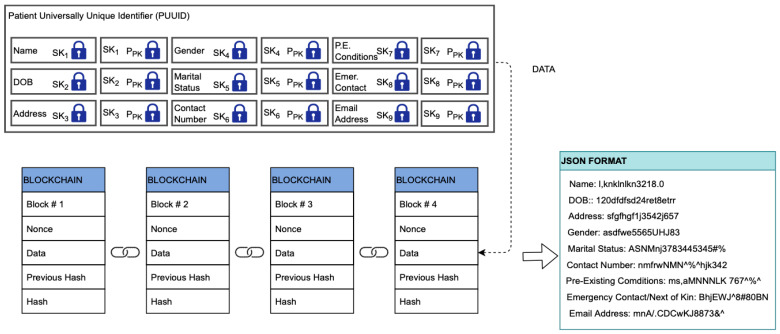
Patient information data structure and PIB commit.

For a patient to be registered on EHRChain, a member of the consortium must be able to collect the relevant patient information and commit it to the PIB. The patient first needs to create an account on the web portal if they do not have one yet by providing an email address and a preferred password. The web portal would then generate a universally unique identifier (UUID), private key, and public key for the patient, which are needed by the consortium member in adding the patient’s information to the PIB. Symmetric keys will also be generated to encrypt each parent information key–value pair, which itself will be encrypted using the patient’s public key. The information required and the process of committing it to the blockchain are illustrated in Figure 11.

**Figure 12:**
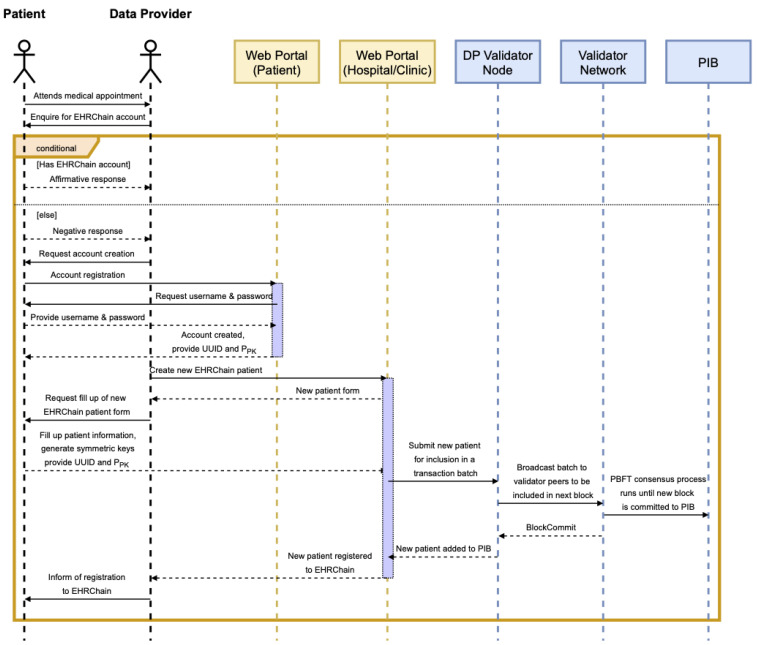
Patient registration sequence diagram.

**Figure 13:**
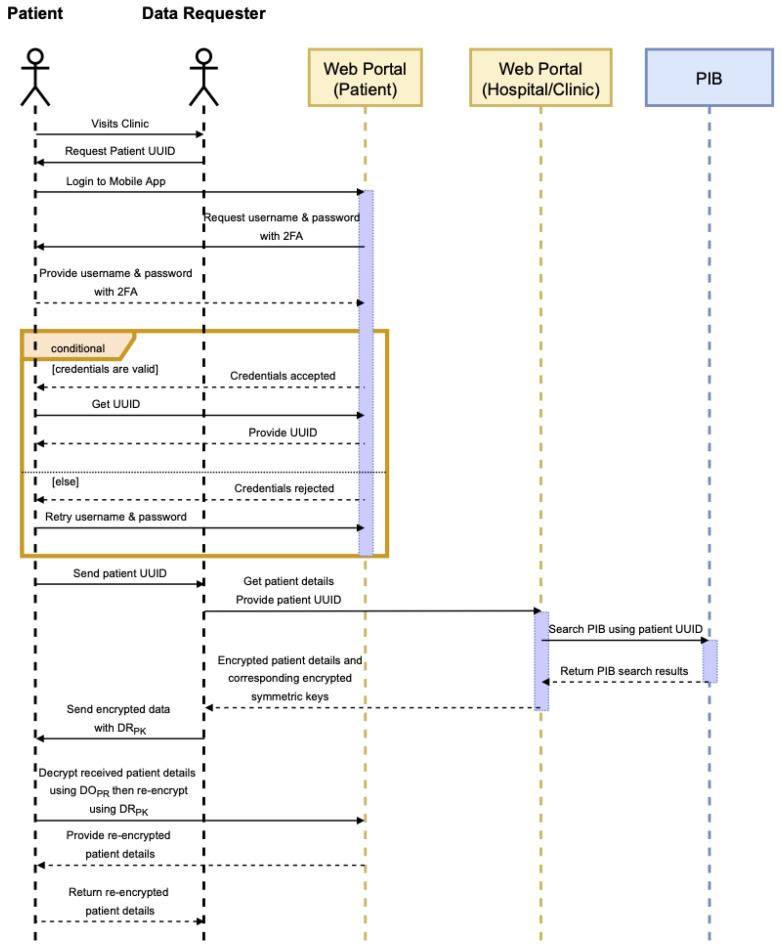
Patient verification sequence diagram.

Once a record for the patient has been validated and added to the PIB, patients as the data owners are able to access their data and make modifications as required. This is performed through the web portal, where patients can use their UUID to locate their record on the blockchain, use their private key to decrypt the symmetric key for that specific line item, and then use the symmetric to re-encrypt the modified value. Patient information is stored as key–value pairs and encrypted individually to aid with patient privacy and confidentiality. When a medical practitioner requires to view specific information about the patient, they can request and view only the line items that they need instead of accessing the complete patient record. This process is illustrated in Figure 12 and Figure 13.

#### 3.2.2. EHR Request and Data Dispensation

**Figure 14:**
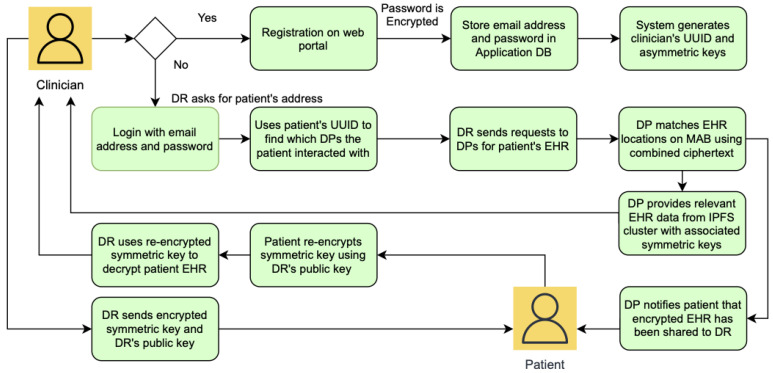
Data requester and EHR distribution flow diagram.

When a patient receives medical services from more than one healthcare provider, it would mean the patient’s health records do not reside with a single organization. It could be the case that the patient deals with more than one member of the consortium or that they receive care from an organization that is not a member of the consortium at all. In either case, the new healthcare provider would need to request the consortium members whom the patient has dealt with in the past for a copy of the patient’s EHR, which would give them a comprehensive view of the patient’s healthcare history. This request process is illustrated in Figure 14.

Similar to patients, a DR also needs to create an account on the web portal if they do not have an account yet—this is so they can generate their own private and public keys. This process would also generate their DP UUID because once a DR generates an EHR for a patient, that means they would be a DP in the future when another DR asks for that specific patient’s EHR.

**Figure 15:**
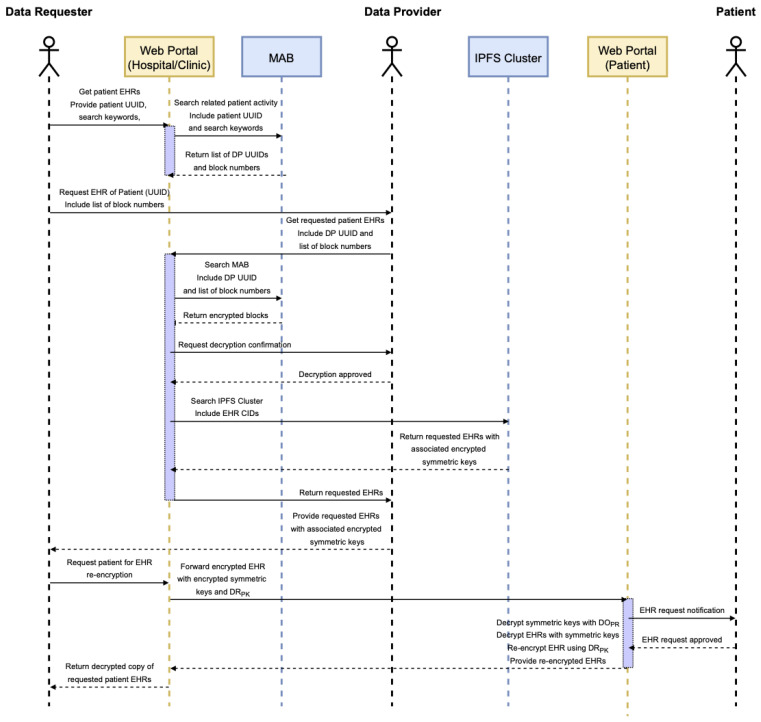
EHR request fulfillment sequence diagram.

After creating an account, a DR can scan the MAB using the patient’s UUID to figure out all the healthcare providers that hold a copy of the patient’s EHR. Once the requester has this information, they can then send the requests for the patient’s EHR. After receiving a request, a DP can safely transmit the encrypted EHR and corresponding symmetric keys to the DR as it requires the patient to re-encrypt to symmetric keys for the DR to be able to decrypt the EHR. To do this, the DR sends the encrypted symmetric keys to the patient together with their public key. The patient can then use this to re-encrypt the symmetric keys using the web app. This process is illustrated in a sequence diagram shown in Figure 15.

#### 3.2.3. EHR Creation and Blockchain Storage

**Figure 16:**
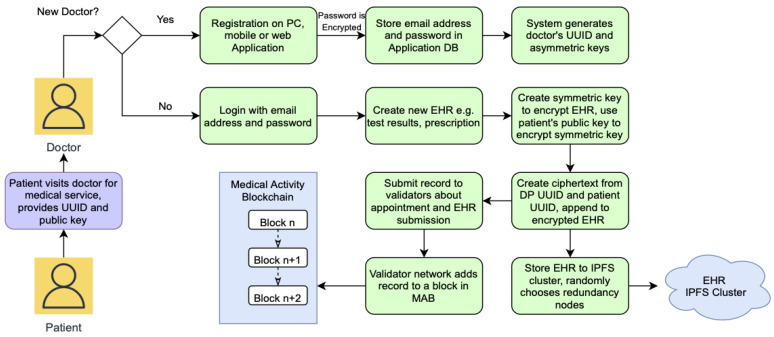
Data provider EHR creation and commit process diagram.

Similar to patients and DRs, DPs also need an account on the web portal. This is a given for consortium members who would need to interact with the MAB and PIB. After a medical appointment with a patient, a healthcare provider presumably generates EHR relating to the patient. Now to commit this record to the blockchain, the healthcare professional attending to the patient uses the web portal to submit the records to their organization’s validator node or nodes. The details of how validators function is described in the next section. The commitment process is illustrated in Figure 16.

**Figure 17:**
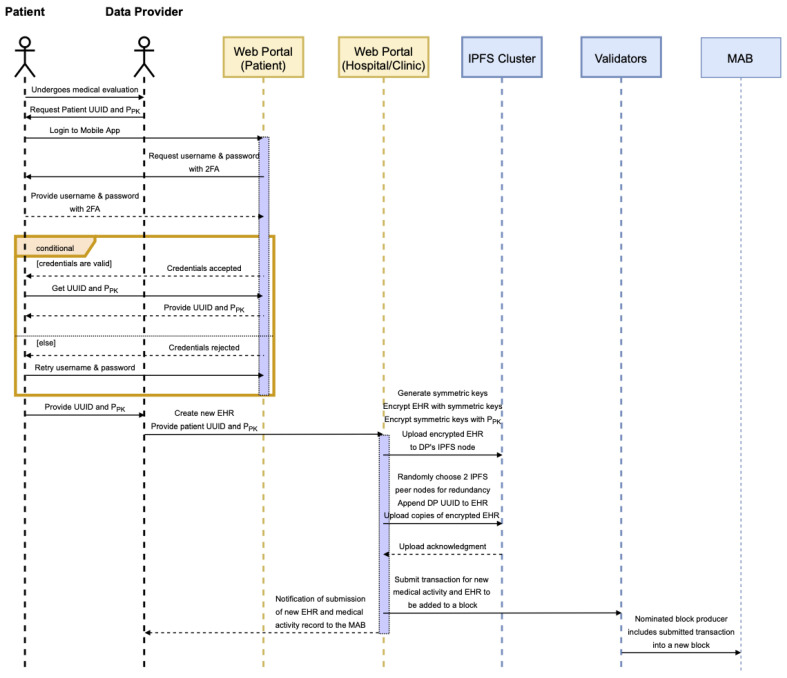
EHR creation and storage sequence diagram.

This process is better explained using a sequence diagram shown in Figure 17.

### 3.3. EHRChain Data Structures

**Figure 18:**
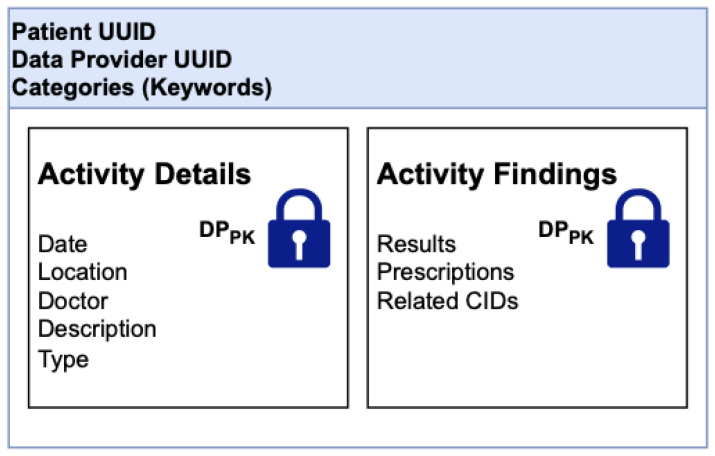
MAB data structure.

**Figure 19:**
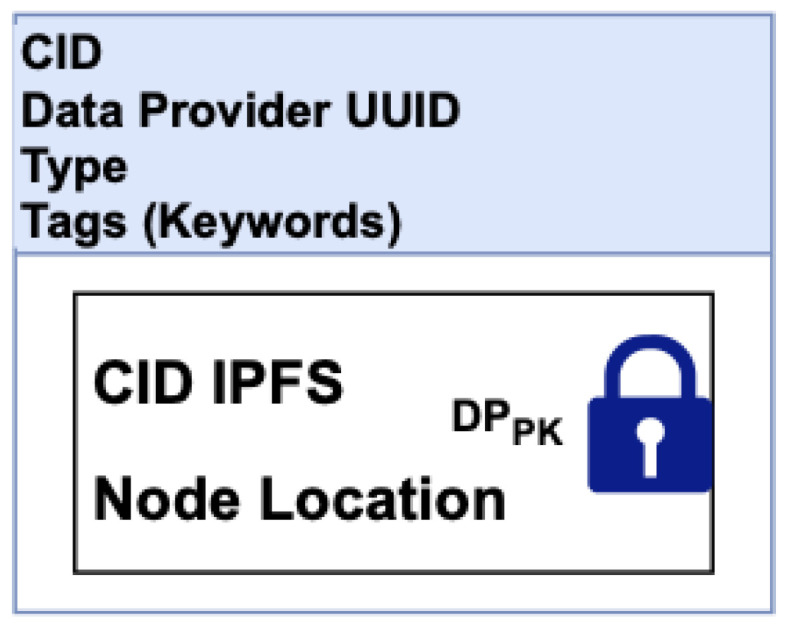
EHR IPFS directory.

The commit process for adding records to the blockchains is better described in Section 3.4, which relates to the design and algorithms of Hyperledger Sawtooth. For clarity, the data structures of the records in the MAB, as well as the EHR IPFS Directory for a data provider, are illustrated in Figure 18 and Figure 19. In Figure 18, this structure allows the searching of the MAB for medical activity relating to a specific patient, a specific DP, or categories that would classify the medical activity. Beyond that, a DR needs the approval of the DP for them to decrypt the records. Figure 19 shows the EHR IPFS directory data structure. This allows DPs to track and search for EHR within IPFS clusters as well as on other IPFS nodes for redundant copies.

**Figure 20:**
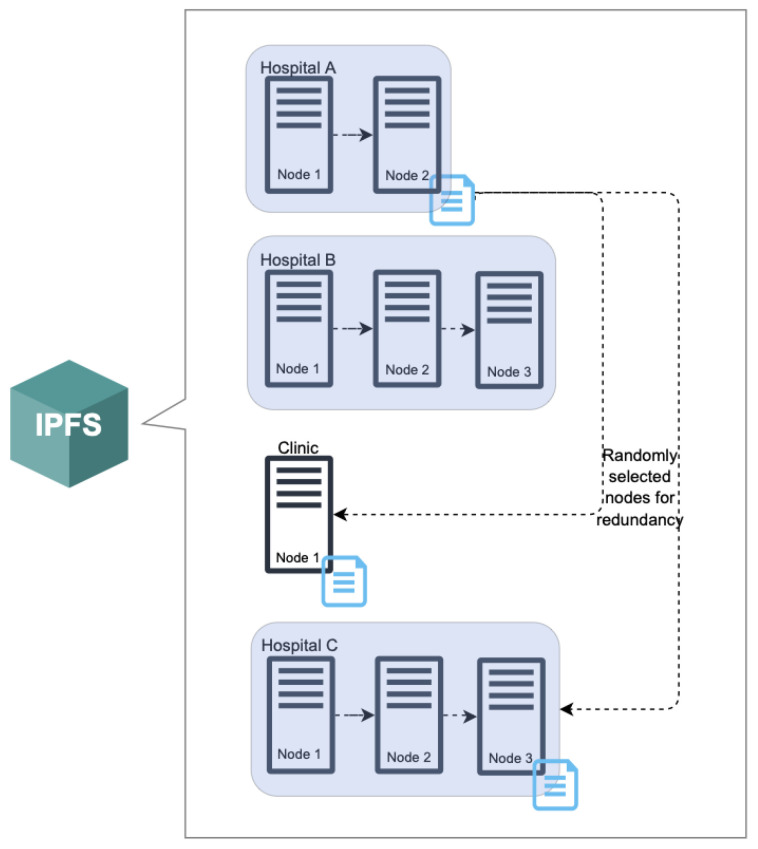
IPFS redundancy for EHRChain.

**Figure 21:**
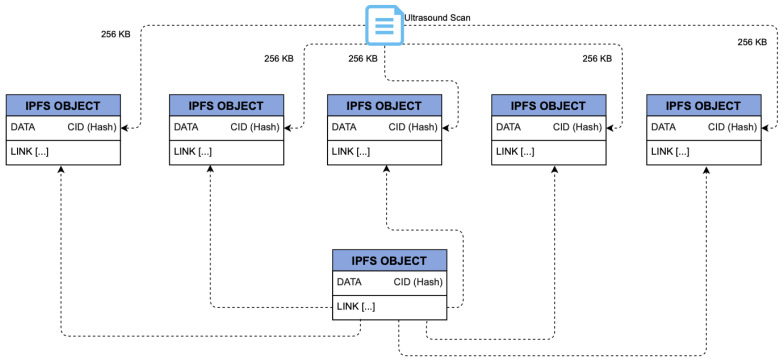
IPFS storage process.

With the rise of ransomware attacks on healthcare organizations, this proposed framework has included data redundancy by storing EHR records on multiple IPFS nodes spread across a number of consortium members, as shown in Figure 20. If a DP experiences an attack that compromises data on its nodes and prevents them from accessing EHR data, that organization has the ability to wipe everything and start from scratch. Medical activity and patient information could be retrieved by downloading the MAB and PIB blockchains. Then, using the records from the dual blockchains, the organization is able to trace and retrieve backups of EHR files from redundant IPFS copies. Figure 21 shows the IPFS storage process to better understand how IPFS saves files.

### 3.4. Committing Healthcare Data to the MAB and PIB

Hyperledger Sawtooth was chosen for the blockchain implementation because of its properties, as described in Section 3.1, with some minor modifications to adapt it for use in EHRChain. One other desirable feature of Hyperledger Sawtooth is pluggable consensus engines, and fortunately, the developers have provided the Sawtooth PBFT algorithm, which provides transaction immutability. This property enhances EHRChain by providing security and data integrity.

The PBFT algorithm is a voting-based algorithm with Byzantine fault tolerances ensuring safety and liveliness. As with other PBFT consensus algorithms, the EHRChain network can tolerate up to a third of malfunctioning or malicious nodes and still be considered secure. Nodes in the network run as validators, sending many messages back and forth to reach consensus, commit blocks, and maintain a healthy leader node called the primary node.

**Figure 22:**
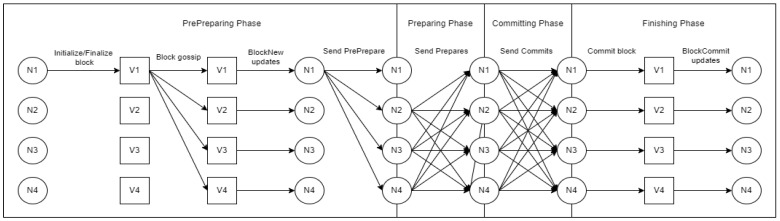
Hyperledger Sawtooth PBFT algorithm phases.

The network switches to a primary node, called a view change, at regular intervals as well as when a majority of the nodes determine that the primary node is faulty. Sawtooth PBFT runs on each node in the network as a consensus engine. The primary node builds and publishes blocks while other nodes, called secondary nodes, vote on blocks and the health of the primary node. The phases for committing a health record to the blockchain are shown in Figure 22 and described in detail after.

1. A doctor uses a client application (e.g., Web Portal) to submit a batch of related transactions to their own DP validator. The client application communicates with the validator either directly or via the REST API.
2. The validator runs transaction processors to apply business logic to the batch of related transactions.
3. When it is ready, the validator broadcasts the batch to the rest of its peers.
4. From here, the validators in the Sawtooth Network follow the normal mode PBFT consensus during normal operations. All nodes begin in the pre-preparing phase.
5. The primary node (the member of the consortium currently set as the leader of the network for a period of time) N1 will send a request to its validator (software running on the node) to initialize a new block and broadcast it to the network including itself.
6. After receiving the block, all nodes will perform preliminary verification to make sure the block is valid and store the block in their PBFT logs.
7. The primary will then broadcast a preprepare message to all nodes containing four key pieces of information:
  - The ID of the block;
  - The block’s number;
  - The primary’s view number;
  - The primary’s ID.
8. When the other nodes receive the preprepare message, it will validate the message (verify the digital signature, check that the view number matches the node’s current view number, and the message came from the right node) and add it to its internal log.
9. Nodes will then move on to the preparing phase. All secondary nodes (not the primary) will broadcast a prepare message to the rest of the network, including itself. The prepare message contains the following information:
  - The ID of the block;
  - The block’s number;
  - The node’s view number;
  - The node’s ID.
10. Each node will wait until it has received 2f + 1 prepare messages that have the same block ID, block number, and view number from different nodes.

$$\begin{array}{c} n=total\;\#\;of\;nodes\;in\;network\\ f=\frac{n-1}{3}\;\left(Max\;\#\;of\;faulty\;nodes\right) \end{array}$$

**Figure 23:**
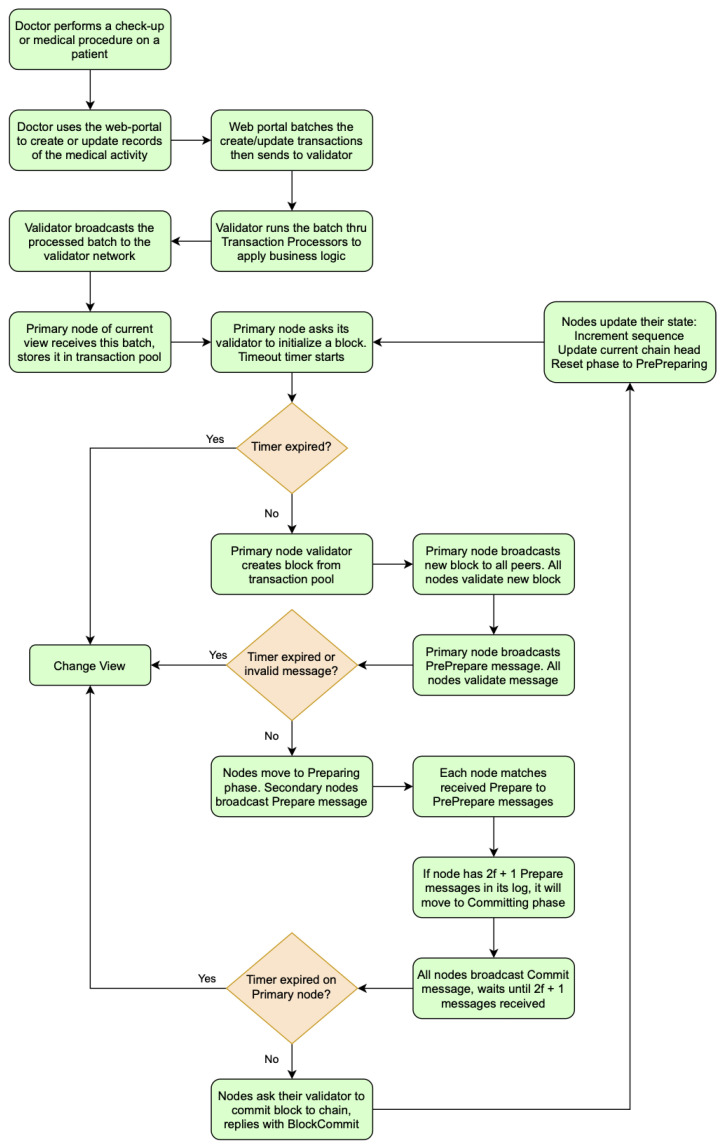
Hyperledger Sawtooth PBFT consensus mechanism process.

11. The nodes then enter the committing phase. All nodes, including the primary, broadcasts a commit message to the whole network, including itself, containing information similar to the previous messages, such as the ID and number of the block along with the node’s view number and ID.
12. Nodes save the received commit messages to their logs. To guarantee that all non-faulty nodes in the network have agreed to commit this block, each node waits to receive 2f + 1 commit message; then, the node moves to the finishing phase.
13. Once in the finishing phase, each node will tell its validator to commit the block for which they have a matching pre-prepare, 2f + 1 prepare messages, and 2f + 1 commit messages.
14. When the validator has successfully committed the block to the chain, it sends a blockcommit notification to the node.
15. After receiving the blockcommit confirmation, the node will update its state as follows:
  - Increment its sequence number by 1;
  - Update its current chain head to the block that was just committed;
  - Reset its phase to pre-preparing.
16. Finally, the primary node will initialize a new block to start the process all over again. The entire process is summarized in a workflow diagram shown in Figure 23.

## 4. Discussion

The systematic review of the existing literature has surfaced the 14 challenges facing existing proposals and implementations related to EHR by combining fragmented objectives across all the reviewed studies, which are: (1) privacy; (2) security; (3) ease of use; (4) confidentiality; (5) interoperability; (6) data ownership; (7) access control; (8) scalability; (9) data validity; (10) authentication; (11) accessibility; (12) data integrity; (13) availability; (14) storage. The reason for this fragmentation is that some challenges are at odds with each other such as privacy and accessibility or security and interoperability. Healthchain was the closest proposal to a complete solution that took 11 out of the 14 challenges into account.

These findings give the current state of research for EHR and provide a roadmap for creating the ideal framework for increasing the adoption and maximizing the potential benefits of EHR. To achieve these stated goals, a criterion that a proposed solution needs to satisfy is the suitability to address all the identified challenges simultaneously. A second criterion is a low barrier to entry for implementing the proposed EHR solution. EHRChain is our attempt to meet these criteria using a framework based on Hyperledger Sawtooth. We note that the list of studies reviewed is not exhaustive; thus, there may be other EHR challenges that were not captured in our results.

EHRChain shows that it is possible to develop a system that can resolve conflicting design objectives inherent with EHR. This is made possible by leveraging advances in the field of cryptography, which gave rise to technologies such as blockchain and IPFS. Our findings, together with our proposed solution, provide a base for future EHR proposals and implementations that intends to approach the digital transformation in the healthcare sector in a more complete manner. Future research could include a simulation of EHRChain to analyze the merits of the proposed framework and could be followed by a proof-of-concept implementation to show the viability of the system’s design.

## 5. Conclusions

In recent years, research into the application of advancements in blockchain technology for electronic health records (EHR) is in full swing to gain its long-promised potential. As blockchain technology matures and researchers better understand the intricacies of the technology, new and creative ways to design EHR systems will emerge. Blockchain technology, which was initially viewed negatively due to its association with cryptocurrencies such as Bitcoin, is now more positively accepted. Another possible cause for this uptake in research using blockchain is the recent cyberattacks that have affected hospitals and other healthcare providers all over the world.

With the advancements in blockchain offerings in the last few years, it is now much easier to prototype, simulate, and launch custom blockchain network implementations that could be applied to EHR. Programmable public blockchains such as Ethereum and permissioned blockchains such as Hyperledger Fabric allow researchers to experiment and explore this technology. Still, applying these solutions in a real-life healthcare setting is difficult. It is promising though that there have been a couple of implementations already, even though these have been performed with quite small organizations.

Aside from blockchain, IPFS has also gained popularity in the quest to improve EHR. It provides decentralized and distributed file storage. IPFS could also help in fighting malicious attacks by making sure there is not a single point of failure for storing these valuable files securely and in a distributed way.

Using Healthchain proposed by Chenthara, S. et al. [37] as an inspiration, the EHRChain framework was designed, which aims to address all 14 EHR challenges that were found in existing proposed solutions. EHRChain is a consortium blockchain based on the Hyperledger Sawtooth platform with a PBFT consensus engine and employs IPFS clusters for health record storage. This framework, however, still needs to go through rigorous testing to ensure it is suitable enough to be implemented on a small scale before moving up to large-scale implementations.

Depending on the result of the tests, the framework design may be complemented or enhanced using other technologies that are also being explored in this field. Lightweight cryptography did not show up in the studies analyzed for systematic review but is also good to be considered since other medical records come from IoT devices where resources are constrained [105]. Proven in other research works is the use of steganography, which helps obscure private information from unauthorized users [106]. This technique can be paired with cryptography and can obtain even better results [107]. Other aspects that also need to be looked at are governance and cost-effectivity of the design.

## Figures and Tables

**Table 1:** Electronic database search.

Electronic Database	Type	URL
CDU	Digital Library	https://www.cdu.edu.au/library (accessed on 4 December 2021)
IEEE Xplore	Digital Library	https://ieeexplore.ieee.org/Xplore/home.jsp (accessed on 4 December 2021)
MDPI	Digital Library	https://www.mdpi.com (accessed on 5 December 2021)
Science Direct—Elsevier	Digital Library	https://www.sciencedirect.com (accessed on 11 December 2021)
Springer	Digital Library	https://www.springer.com/gp (accessed on 6 December 2021)
Wiley online library	Digital Library	https://www.wiley.com/en-au (accessed on 7 December 2021)
Google Scholar	Search Engine	https://scholar.google.com.au (accessed on 9 December 2021)
Researchgate	Social networking site	https://www.researchgate.net (accessed on 10 December 2021)

**Table 2:** Search queries used for the systematic review.

Search Queries	
SQ1	“electronic health record” AND blockchain OR cryptography OR security OR privacy OR decentralise
SQ2	“electronic health record” AND blockchain OR cryptography OR security OR privacy OR distributed file system
SQ3	“personal health record” AND blockchain OR cryptography OR security OR privacy OR decentralise
SQ4	“personal health record” AND blockchain OR cryptography OR security OR privacy OR decentralise

**Table 3:** Inclusion and Exclusion Criteria.

List of Inclusion and Exclusion Criteria	
***Inclusion Criteria (IC)***	
IC1	Should be listed in one of the chosen databases
IC2	Should be published in the last 10 years (2011–2021)
IC3	Should contain at least one of the keywords
IC4	Should be published in a journal, conference, or magazine
IC5	Title, abstract, and full text should match the study being searched for
***Exclusion Criteria (EC)***	
EC1	Duplicate items
EC2	Studies not written in English
EC3	Proposed solution is not designed for EHR nor PHR
EC4	Full text cannot be obtained

**Table 4:** Summary of scores for assessing the quality of papers.

	QE1. Is the Publication Pertaining to EHR or PHR?	QE2. Is the Proposed Solution Well Defined?	QE3. Are the Challenges Being Addressed by Their Proposed Solution Clearly Stated?	QE4. Did the Publication Define the Proposed Solutions’ Limitations?	QE5. Is the Proposed Solution Ready for Implementation?	Summary of Points
1 [37]	4	4	4	4	2	18
2 [38]	4	4	3	0	2	13
3 [39]	4	4	4	4	2	18
4 [40]	4	4	4	0	2	14
5 [41]	4	3	4	3	2	16
6 [42]	4	4	3	0	0	11
7 [43]	4	3	3	2	2	14
8 [44]	4	3	3	3	2	15
9 [45]	4	4	3	2	2	15
10 [46]	4	4	4	3	0	15
11 [47]	4	4	4	4	2	18
12 [48]	4	4	4	3	2	17
13 [49]	4	4	3	3	0	14
14 [50]	4	4	4	4	2	18
15 [51]	4	4	4	2	2	16
16 [52]	4	3	3	3	0	13
17 [53]	4	4	3	3	0	14
18 [54]	4	4	3	4	2	17
19 [55]	4	4	3	3	0	14
20 [56]	4	4	4	2	4	18
21 [57]	4	4	4	0	2	14
22 [58]	4	4	4	4	0	16
23 [59]	4	4	3	3	2	16
24 [60]	4	4	3	2	2	15
25 [61]	4	4	4	2	0	14
26 [62]	4	4	3	0	2	13
27 [63]	4	4	4	2	0	14
28 [64]	4	4	4	4	2	18
29 [65]	4	2	3	2	0	11
30 [66]	4	4	4	1	2	15
31 [67]	4	3	4	3	0	14
32 [68]	4	4	4	0	2	14
33 [69]	4	4	3	4	2	17
34 [70]	4	4	4	2	2	16
35 [71]	4	4	2	3	2	15
36 [72]	4	3	3	1	2	13
37 [73]	4	4	2	0	2	12
38 [74]	4	4	3	4	2	17
39 [75]	4	3	3	4	0	14
40 [76]	4	3	3	2	4	16
41 [77]	4	4	3	2	2	15
42 [78]	4	3	4	4	2	17
43 [79]	4	4	4	0	0	12
44 [80]	4	3	3	3	0	13
45 [81]	4	4	3	4	2	17
46 [82]	4	4	3	2	0	13
47 [83]	4	4	3	4	2	17
48 [84]	4	3	3	2	2	14
49 [85]	4	3	4	2	0	13
50 [86]	4	4	3	0	0	11
51 [87]	4	4	3	4	2	17
52 [88]	4	4	4	3	0	17
53 [89]	4	4	3	4	2	17
54 [90]	4	4	3	4	0	15
55 [91]	4	3	3	4	0	14
56 [92]	4	2	3	2	0	11
57 [93]	4	3	3	0	2	12
58 [94]	4	4	4	1	0	13
59 [95]	4	4	4	0	0	12
60 [96]	4	2	4	1	0	11
61 [97]	4	2	2	2	2	12
62 [98]	4	3	2	1	2	12
63 [99]	4	4	2	1	2	13
64 [100]	4	4	3	4	4	19
65 [101]	4	2	4	1	0	11

**Table 5:** Proposed solutions that address EHR challenges and the technologies used.

	Technologies Used	Types of Blockchain	Proposal (P), Simulated (S), Implemented (I)	Privacy	Security	Confidentiality	Interoperability	Accessibility	Scalability	Availability	Authentication	Access Control	Data Integrity	Data Validity	Data Ownership	Data Storage	Ease of Use	Total
1 [37]	Angular 4, Chaincode, Representational State Transfer (REST) Server (REST API), CouchDB, Fabric Client, Practical Byzantine Fault Tolerance (PBFT), Hyperledger Composer, Hyperledger Fabric, InterPlanetary File System (IPFS)	Permissioned–Private	S	⚫️	⚫️	⚫️	⚫️		⚫️		⚫️	⚫️	⚫️		⚫️	⚫️	⚫️	11/14
2 [38]	Authentication Server, Certification Authority, Permissioned blockchain, Smart Contract	Permissioned–Private	S	⚫️	⚫️	⚫️		⚫️	⚫️	⚫️	⚫️	⚫️			⚫️		⚫️	10/14
3 [39]	Chaincode, CouchDB, Health Insurance Portability and Accountability Act (HIPAA)-compliant cloud storage, Hyperledger Fabric (Membership Service [MS], Certificate Authority [CA], Solution Users [SU]), Orderer, PKI-based asymmetric encryption, Web Application	Permissioned	S	⚫️	⚫️	⚫️	⚫️		⚫️	⚫️		⚫️	⚫️			⚫️		9/14
4 [40]	Cloud storage, Hash table, Hyperledger Fabric	Permissioned–Private	S	⚫️	⚫️	⚫️		⚫️	⚫️	⚫️		⚫️	⚫️			⚫️		9/14
5 [41]	Ateniese, Fu, Green, and Hohenberger Proxy Re-Encryption (AFGH PRE), Cloud storage, Hyperledger Fabric	Permissioned–Private	S	⚫️	⚫️	⚫️				⚫️	⚫️	⚫️	⚫️		⚫️	⚫️		9/14
6 [42]	Cloud storage, Hyperledger Fabric, Proxy Re-encryption	Permissioned–Private	P	⚫️	⚫️	⚫️				⚫️		⚫️	⚫️		⚫️	⚫️	⚫️	9/14
7 [43]	Ethereum, IPFS, Proof of Authority (PoA), Smart contract, Certificate Authority (CA)	Public	S	⚫️	⚫️		⚫️		⚫️	⚫️	⚫️	⚫️	⚫️			⚫️		9/14
8 [44]	Blockchain, Keyless Signature Infrastructure (KSI)	Not Defined	S	⚫️	⚫️	⚫️			⚫️	⚫️	⚫️	⚫️	⚫️				⚫️	9/14
9 [45]	Hyperledger Fabric, REST services	Permissioned–Private	S	⚫️	⚫️		⚫️	⚫️	⚫️		⚫️	⚫️				⚫️	⚫️	9/14
10 [46]	Consortium Blockchain, Ethereum, Proof of Authorisation, Smart Contracts	Public Permissioned–Consortium	P	⚫️	⚫️	⚫️				⚫️	⚫️	⚫️	⚫️		⚫️	⚫️		9/14
11 [47]	Consortium blockchain, Public-Coin Chameleon Hashing (PCCH), Smart Contracts, Proxy Re-Encryption (PRE), 2-party PRE decryption (2PD)	Permissioned–Consortium	S	⚫️	⚫️	⚫️	⚫️		⚫️		⚫️		⚫️		⚫️			8/14
12 [48]	Attribute-Based Signcryption Algorithm (ABSC), Cloud Storage, Ethereum	Public	S	⚫️	⚫️	⚫️		⚫️			⚫️		⚫️	⚫️		⚫️		8/14
13 [49]	Ethereum, IPFS, Smart Contracts	Public	P	⚫️	⚫️	⚫️			⚫️			⚫️	⚫️			⚫️	⚫️	8/14
14 [50]	Ethereum, IPFS, Multi-Party Authorization (MPA), Reputation-governed Trusted Oracles (RGTO), Smart Contracts	Public	S	⚫️	⚫️	⚫️				⚫️		⚫️	⚫️		⚫️	⚫️		8/14
15 [51]	Burrows–Abadi–Needham (BAN) logic analysis, Cloud computing, Hyperledger Fabric	Permissioned–Private	S	⚫️	⚫️				⚫️		⚫️	⚫️	⚫️	⚫️		⚫️		8/14
16 [52]	Ethereum blockchain, Smart contracts	Private	P	⚫️	⚫️	⚫️		⚫️	⚫️			⚫️	⚫️		⚫️			8/14
17 [53]	Attribute-Based Encryption (ABE), Blockchain, Identity-Based Encryption (IBE), Identity-based Signature (IBS)	New Design	P	⚫️	⚫️	⚫️	⚫️				⚫️	⚫️	⚫️			⚫️		8/14
18 [54]	Amazon cloud, Ethereum, IPFS, Mobile app, Smart Contracts	Public	S	⚫️	⚫️					⚫️	⚫️	⚫️	⚫️		⚫️	⚫️		8/14
19 [55]	Ethereum, Smart Contracts, Symmetric Searchable Encryption	Public	P	⚫️	⚫️	⚫️	⚫️			⚫️		⚫️	⚫️		⚫️			8/14
20 [56]	Modex Blockchain Database (BCDB), MongoDB, Permissioned blockchain, Tendermint	Permissioned–Private	I	⚫️	⚫️	⚫️	⚫️								⚫️	⚫️	⚫️	7/14
21 [57]	Delegated Proof of Stake (DPoS) consensus mechanism, Proxy Re-Encryption (PRE), Private Blockchain (also used as storage)	Permissioned–Private	S	⚫️	⚫️			⚫️			⚫️	⚫️			⚫️	⚫️		7/14
22 [58]	Consortium Blockchain, Ethereum, Off-chain Blockchain Systems (OCBS), Proof of Authority (PoA)	Public Permissioned–Consortium	P	⚫️	⚫️		⚫️	⚫️	⚫️						⚫️	⚫️		7/14
23 [59]	Certificate Authority, Kafka Ordering Consensus Mechanism (Orderers, Apache Kafka, Zookeeper), Hyperledger Fabric, Smart Contract	Permissioned	S	⚫️		⚫️			⚫️		⚫️	⚫️	⚫️	⚫️				7/14
24 [60]	Cloud storage, Ethereum, Smart contract, Searchable encryption	Public	S		⚫️	⚫️					⚫️		⚫️	⚫️	⚫️	⚫️		7/14
25 [61]	Cyphertext Policy Attribute-Based Encryption (CP-ABE), Ethereum, Smart Contracts	Public	P	⚫️	⚫️	⚫️						⚫️	⚫️		⚫️		⚫️	7/14
26 [62]	Attribute-Based Encryption (ABE), Attribute-Based Multi-Signature (ABMS), Hyperledger Fabric, Hyperledger Ursa Library, Off-chain edge node	Permissioned–Private	S	⚫️	⚫️				⚫️		⚫️	⚫️			⚫️	⚫️		7/14
27 [63]	Ethereum, Smart Contracts, RPM (Remote Patient Monitoring), IoT, Django (python), PoA (proof of authority)	Public but permissioned via PoA	P	⚫️	⚫️	⚫️		⚫️			⚫️		⚫️				⚫️	7/14
28 [64]	Hyperledger Fabric, Hyperledger Composer, Hyperledger Caliper	Permissioned–Private	S	⚫️	⚫️		⚫️		⚫️			⚫️	⚫️			⚫️		7/14
29 [65]	Ethereum, Cloud Storage (Data Lake), smart contracts	Public	P	⚫️	⚫️			⚫️			⚫️	⚫️			⚫️	⚫️		7/14
30 [66]	Distributed applications, Ethereum, Hyperledger Fabric, PoW (Proof of Work) consensus mechanism, Smart contracts	PublicPermissioned–Private	S	⚫️	⚫️	⚫️	⚫️			⚫️			⚫️					6/14
31 [67]	Blockchain (not specified), Smart contracts	Permissioned	P	⚫️	⚫️	⚫️					⚫️		⚫️		⚫️			6/14
32 [68]	Certificateless Aggregate Signature scheme (CAS), Elliptic Curve Cryptography (ECC), Ethereum, CSP (Cloud Service Provider)	Public	S	⚫️	⚫️	⚫️							⚫️	⚫️		⚫️		6/14
33 [69]	Blockchain, Chord algorithm, Publish-Subscribe system, Routing Overlay	New Design	S	⚫️	⚫️		⚫️	⚫️	⚫️					⚫️				6/14
34 [70]	Cloud storage, Ethereum	Public	S	⚫️	⚫️	⚫️					⚫️		⚫️			⚫️		6/14
35 [71]	Blockchain, Deep Learning, Ring Learning with Error (RLWE) lattice-based cryptography	Not Defined	S	⚫️	⚫️	⚫️			⚫️		⚫️				⚫️			6/14
36 [72]	Ethereum	Public	S	⚫️	⚫️						⚫️	⚫️		⚫️	⚫️			6/14
37 [73]	Ethereum, IPFS, Permissioned Blockchain, Proof of Authority (PoA), Smart Contracts	Permissioned–Private	S	⚫️	⚫️							⚫️		⚫️	⚫️	⚫️		6/14
38 [74]	Distributed Ledger, Hyperledger Fabric, Idemix	Permissioned–Private	S	⚫️	⚫️	⚫️			⚫️			⚫️		⚫️				6/14
39 [75]	Ethereum, IPFS	Public	P	⚫️	⚫️	⚫️						⚫️			⚫️	⚫️		6/14
40 [76]	Identity as a stake consensus mechanism, Permissioned blockchain (also used as storage), Proof of Authority (PoA)	Permissioned–Private	I	⚫️	⚫️			⚫️				⚫️		⚫️		⚫️		6/14
41 [77]	Hyperledger Fabric	Permissioned–Private	S		⚫️				⚫️			⚫️	⚫️				⚫️	5/14
42 [78]	Ethereum, IPFS, PRE, Trusted Oracles and Reputation System, Smart Contracts	Public	S		⚫️		⚫️	⚫️				⚫️				⚫️		5/14
43 [79]	Blockchain (also used as storage), IoT, Mobile devices	Not Defined	P	⚫️	⚫️			⚫️			⚫️					⚫️		5/14
44 [80]	AI based intelligent agents, Blockchain (DLT), Smart Contract	Not Defined	P	⚫️	⚫️						⚫️		⚫️		⚫️			5/14
45 [81]	Attribute-Based Encryption (ABE), Advanced Encryption Standard (AES), Distributed Hash Table, Ethereum, IPFS, SC-ABSE (CP-ABE + SSE + smart contract)	Public	S	⚫️	⚫️	⚫️						⚫️				⚫️		5/14
46 [82]	Consortium Blockchain, Private Blockchain, Proof of Conformance	Permissioned–PrivatePermissioned–Consortium	P	⚫️	⚫️	⚫️				⚫️		⚫️						5/14
47 [83]	Practical Byzantine Fault Tolerance (PBFT), Permissioned Blockchain	Permissioned–Private	S	⚫️	⚫️	⚫️							⚫️	⚫️				5/14
48 [84]	Attribute-Based Encryption (ABE), Blockchain. Cloud storage	Not Defined	S	⚫️	⚫️							⚫️	⚫️			⚫️		5/14
49 [85]	Attribute-Based Proxy Re-Encryption (ABPRE), Cloud storage	Not Specified/Own design	P		⚫️							⚫️	⚫️	⚫️		⚫️		5/14
50 [86]	Blockchain, delegated Byzantine Fault Tolerance (dBFT) consensus algorithm	Not Defined	P	⚫️	⚫️			⚫️			⚫️	⚫️						5/14
51 [87]	Blockchain-based data storage, Decentralising Attribute-Based Signature (DABS), Practical Byzantine Fault Tolerance (PBFT) consensus mechanism	Not Defined	S	⚫️	⚫️						⚫️		⚫️			⚫️		5/14
52 [88]	Advanced cryptographic techniques, Ethereum, Smart Contracts, PRE	Permissioned	P	⚫️	⚫️		⚫️					⚫️	⚫️		⚫️		⚫️	5/14
53 [89]	Blockchain, Hash Table, IPFS, Machine Learning Unit, Smart contract, Smart device	Not Defined	S	⚫️	⚫️						⚫️		⚫️			⚫️		5/14
54 [90]	Ethereum, IPFS, Smart Contract	Public	P	⚫️	⚫️							⚫️			⚫️	⚫️		5/14
55 [91]	Ethereum, Smart Contracts	Public	P	⚫️	⚫️		⚫️						⚫️		⚫️			5/14
56 [92]	Ethereum, Hyperledger Fabric, Machine Learning	PublicPermissioned–Private	P	⚫️	⚫️			⚫️				⚫️						4/14
57 [93]	Asynchronous BFT (ABFT) consensus mechanism, NPoS (Nominated Proof of Stake), Polkadot Relay Chain, CSP (Cloud Service Provider)	Public	S	⚫️	⚫️				⚫️							⚫️		4/14
58 [94]	Blockchain, MA-ABS (Multi Authority–Attribute-Based Signature)	New Design	P	⚫️	⚫️									⚫️	⚫️			4/14
59 [95]	Cipher text-based attribute encryption, Blockchain, Cloud	Permissioned	P	⚫️	⚫️						⚫️	⚫️						4/14
60 [96]	Hyperledger Fabric	Permissioned–Private	P	⚫️	⚫️							⚫️			⚫️			4/14
61 [97]	Hyperledger Fabric, CA	Permissioned Consortium	S	⚫️	⚫️							⚫️					⚫️	4/14
62 [98]	Blockchain handshaker, Cloud, Public blockchain network (blockchain nodes, distributed ledger, and smart contracts), and User application	Public	S		⚫️								⚫️			⚫️		3/14
63 [99]	Blockchain and Distributed Ledger Based Improved Bio-Medical Security System (BDL-IBS), Blockchain, Distributed Ledger	New Design	S	⚫️	⚫️						⚫️							3/14
64 [100]	Hyperledger Fabric, IPFS, Distributed Applications, Smart Contracts	Permissioned	I	⚫️	⚫️		⚫️		⚫️				⚫️					3/14
65 [101]	Health Records Blockchain System (HRBS), Identity Blockchain System (IBS)	New Design	P			⚫️						⚫️						2/14
				59	63	32	16	15	22	13	30	42	38	13	27	37	11	

⚫️—symbol used to mark that the challenge exists on that paper.

**Table 6:** How to address all the challenges found in proposed solutions.

Challenge	Proposed Solution
Privacy	Patient information and medical activity are recorded on separate blockchains, while health records are stored in IPFS.
Security	PBFT consensus ensures security as long as not more than 1/3 of the validators are malicious.
Confidentiality	Patient information, medical activity, and health records are all encrypted via public-key cryptography.
Interoperability	Non-media files should be stored in JSON format to allow different applications to build compatibility independent of other applications
Accessibility	Data requesters (DR) are not required to be part of the consortium to submit a request.
Scalability	At any given period of time (epoch), a subset of validators is chosen to produce blocks to increase throughput.
Availability	Because all medical data are distributed either on blockchains or IPFS, data is available as long as the majority of nodes are online.
Authentication	System users have username and password to access applications as well as private and public keys to encrypt/decrypt data from blockchain/IPFS.
Access Control	Hyperledger Sawtooth provides fine-grained permissions to allow data owners (DO) to control access to their EHR data.
Data Integrity	True immutability due to distributed architecture ensures EHR data cannot be tampered with when the supermajority of nodes is honest.
Data Validity	DO and data providers (DP) need to sign off before EHR data is committed to the blockchain or uploaded to IPFS.
Data Ownership	DO needs to approve by decrypting or by creating the re-encryption key and sending it back to the DR.
Data Storage	EHR data is stored on IPFS, and redundant copies are distributed to consortium members to serve as a backup in case of ransomware attacks.
Ease of Use	Since Hyperledger Sawtooth core is separate from application layer; desktop, web, and mobile applications can be developed independently.

## Data Availability

Not applicable.
